# Pseudomonas Necrotizing Fasciitis in an Otherwise Healthy Infant

**DOI:** 10.1155/2012/517135

**Published:** 2012-11-06

**Authors:** Shakeel Ahmed, Syed Rehan Ali, Zahra Aziz Samani

**Affiliations:** Department of Paediatrics and Child Health, Aga Khan University Hospital, Stadium Road, P.O. Box 3500, Krachi 74800, Pakistan

## Abstract

Necrotizing fasciitis is an uncommon rapidly progressing infection of soft tissue characterized by a severe inflammation of the fascia and soft tissue. The disease is associated with necrosis and subcutaneous gangrene of the inflamed tissue with systemic toxicity that carries a significant mortality unless timely diagnosed and aggressively treated. Monomicrobial necrotizing fasciitis caused by *Pseudomonas aeruginosa* is an exceptionally uncommon condition with only few cases reported in the literature so far. We are reporting a six-month-old female infant who was previously healthy and who presented with necrotizing fasciitis and isolates *Pseudomonas aeruginosa* both from the blood and tissue. The child improved after the intensive treatment.

## 1. Introduction

Necrotizing fasciitis (NF) is a severe soft tissue potentially fatal bacterial infection characterized by rapid progressing necrosis involving mainly the fascia and subcutaneous tissue but can also extend to involve muscles and skin [1]. This rare, life-threatening condition has been recognized since the fifth century BC [2]. All age groups, including neonates, can be affected [3]. Reported incidence in the literature has been described as 0.08 per 100000 children per year with most lesions reported on the trunk [4]. It frequently affects a previously healthy children [5] and results in a significant rate of mortality as well as morbidity if there is any delay in diagnosis and treatment [6]. Accurate early diagnosis and surgical intervention combined with administration of appropriate parenteral antibiotics have been the cornerstones of NF treatment [7].

We are reporting here a case of a six-month-old female infant who was previously well, diagnosed as necrotizing fasciitis secondary to *Pseudomonas aeruginosa*—rare cause of NF in an otherwise healthy infant.

## 2. Case Report

**Figure 1:**
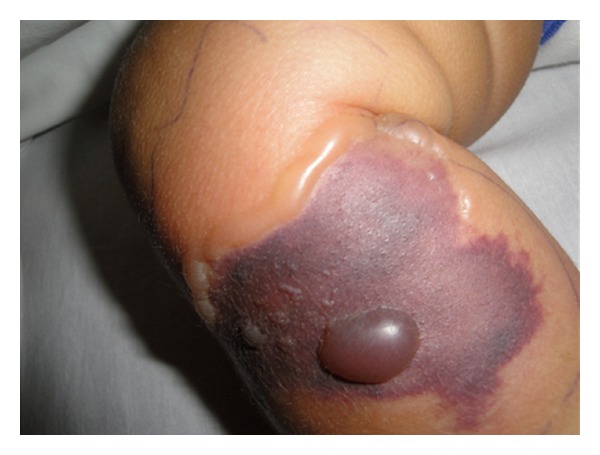
Local lesion with dark bluish black patch with well-circumscribed margins, surrounding erythema with edema, and blisters.

**Figure 2:**
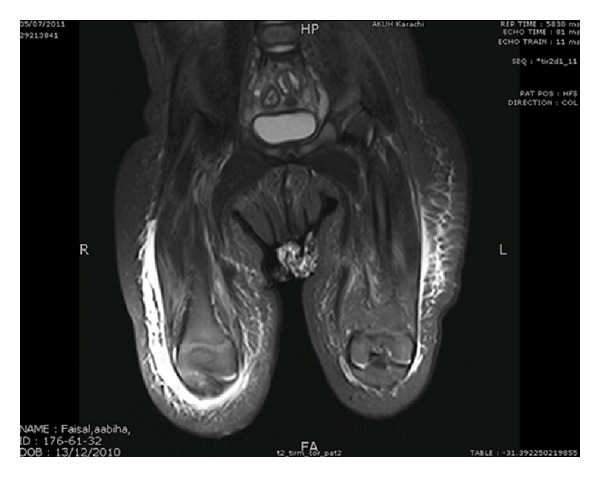
MRI showed extensive edema involving skin, subcutaneous tissue, and deep fascia with multiple areas of negative enhancement in the deep subcutaneous fascia representing small collections.

**Figure 3:**
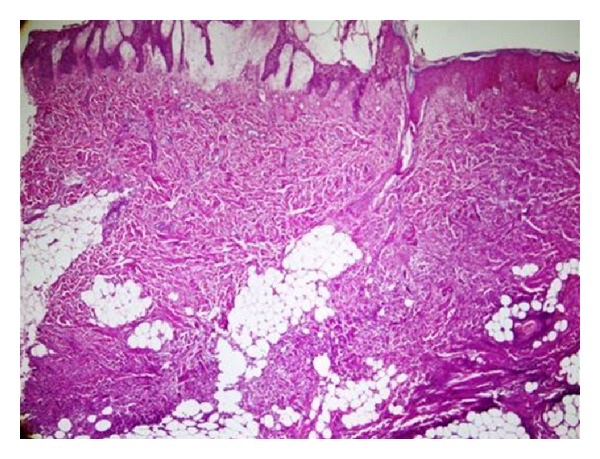
Histopathology shows subepidermal bullus formation with dense dermal acute inflammation extending into the dermal fat.

A six-month-old female infant presented with the 7 days history of high fever and rashes over both thighs, visiting from USA since birth and was previously well. The birth and developmental history were unremarkable and she was fully vaccinated accordingly. There was no history of apparent predisposing factor including drug or insect bite. Examination revealed a well thriving, febrile, irritable child with pulse of 100 beats per minute, maintaining saturation in room air and was normotensive (90/60 mmHg). Local examination revealed erythema fulminans measuring 5 × 5 cm over right leg and left thigh (3 × 5 cm) with well circumscribed margins and surrounding blisters (Figure 1). These lesions were tender and warm on palpation. Rest of the systems including central nervous system was unremarkable. Initial diagnosis necrotizing fasciitis with sepsis was made. Vancomycin (60 mg/kg/24 hr I/V in 4 divided doses) and meropenem (120 mg/kg/24 hr I/V in 3 divided doses) were started. Laboratory workup showed low hemoglobin of 9 gm% and high leukocyte count of 27.8 × 10^9^/L with predominant neutrophil (82%). C-reactive protein was raised at 13.8 U/L. Blood culture grew *Pseudomonas aeruginosa* sensitive to meropenum. X-ray thigh showed extensive hyperemic changes in the soft tissues with blurring of the intermuscular plains. Magnetic resonance imaging (MRI) lower limbs revealed extensive edema of skin, subcutaneous tissue, and deep fascia. There were multiple areas of negative enhancement in the deep subcutaneous fascia of both lower limbs representing small collections. Hyperemic changes within the muscles showing mild enhancement but no definite evidence of osteomyelitis (Figure 2). Incision and debridement of the lesions were performed with removal of the necrotic tissue. Drains were placed on both thighs which were removed after 5 days. Tissue culture also grew *Pseudomonas aeruginosa* which was sensitive to meropenum and ceftazidime. Histopathology of the tissue confirms the diagnosis of necrotizing fasciitis (Figure 3). i.v. meropenum was continued for three weeks along with daily wound care. The child was discharged subsequently in a stable state with planning of skin graft in the future.

## 3. Discussion

Necrotizing fasciitis is a rare, rapidly progressive and potentially fatal infection of the superficial fascia and subcutaneous cellular tissue. Clinically, it is characterized by a massive destruction of tissue; it is usually accompanied by systemic signs of toxicity; the condition has high rate of mortality and morbidity [3].

*Pseudomonas aeruginosa* can be a fatal cause of necrotizing fasciitis [8–10]. NF has been reported in 0.08 per 100000 children per year with most lesions reported on the trunk [4]. Other studies have reported the lesion to occur on the trunk/back as well [3]; however, our patient had the lesion on both lower limbs. The predisposing factors are hematological malignancy, diabetes mellitus, and infancy in majority of the cases [9].

There are two main groups of necrotizing fasciitis depending on microbiology [1]. Type I NF is polymicrobial and most common bacterial species include Gram-positive cocci, enterococci, and Gram-negative Enterobacteriaceae [1]. Type II infections are monomicrobial and usually caused by group A *Streptococcus* either alone or in association with *Staphylococcus aureus* [1]. Type II NF is far less common than type I infection and tends to occur in otherwise healthy, young, immunocompetent hosts and is classically located on the extremities [1].

The most common early signs are erythema, local warmth, skin induration, and edema [1]. These signs often make early diagnosis difficult and the condition is often diagnosed as cellulites and the diagnosis of necrotizing fasciitis is only suspected when the patient fails to respond to broad spectrum intravenous antibiotics or develop cutaneous manifestations [1].

Our patient had no identifiable trauma or apparent predisposing factor and this is consistent with a study done by Childers et al. where nearly seventeen per cent of the patients showed no identifiable antecedent trauma [11]. 

Aggressive surgery and debridement are usually required in combination with antibiotic therapy to limit the spread of the infection [12]. However, early recognition is complicated by the difficulty in distinguishing it from other, less serious soft-tissue infections such as cellulitis, which presents with similar findings of erythema, swelling, and pain. The identification of the key differences in presentation between necrotizing fasciitis and cellulitis is essential for early and effective management [12]. Magnetic resonance imaging (MRI) has the highest sensitivity (93–100%) for diagnosing necrotizing fasciitis. NF exhibits high signal intensity on T2-weighted images by MRI with hyperintense signal corresponding to fluids associated with NF [5]. 

Treatment of NF includes appropriate injectable antibacterial therapy, prompt and aggressive exploration, and debridement of suspected deep seated infection, and supportive measures for the management of shock and multiorgan failure [13]. 

## 4. Conclusion

NF is a life-threatening condition. Its early and correct diagnosis is important in order to improve the outcome. Antibiotics and surgical debridement are important parts of its management.

## References

[1] Naqvi GA, Malik SA, Jan W (2009). Necrotizing fasciitis of the lower extremity: a case report and current concept of diagnosis and management. *Scandinavian Journal of Trauma, Resuscitation and Emergency Medicine*.

[2] Descamps V, Aitken J, Lee MG (1994). Hippocrates on necrotising fasciitis. *The Lancet*.

[3] Pandey A, Gangopadhyay AN, Sharma S, Kumar V, Gopal S, Gupta D (2009). Surgical considerations in pediatric necrotizing fasciitis. *Journal of Indian Association of Pediatric Surgeons*.

[4] Fustes-Morales A, Gutierrez-Castrellon P, Duran-McKinster C, Orozco-Covarrubias L, Tamayo-Sanchez L, Ruiz-Maldonado R (2002). Necrotizing fasciitis: report of 39 pediatric cases. *Archives of Dermatology*.

[5] Abass K, Saad H, Abd-Elsayed AA (2008). Necrotizing fasciitis with toxic shock syndrome in a child: a case report and review of literature. *Cases Journal*.

[6] Surahio AR, Khan AA, Farooq MU, Fatima I, Azhar MZ (2009). Prevalence of necrotizing fasciitis during Ramadan and Hajj 1427-H. *Journal of Ayub Medical College Abbottabad*.

[7] Lee CY, Kuo LT, Peng KT, Hsu WH, Huang TW, Chou YC (2011). Prognostic factors and monomicrobial necrotizing fasciitis: gram-positive versus gram-negative pathogens. *BMC Infectious Diseases*.

[8] Abada A, Benhmidoune L, Tahiri H (2007). Necrotizing fasciitis caused by pseudomonas aeruginosa (an obervation). *Bulletin de la Société belge d’ophtalmologie*.

[9] Akamine M, Miyagi K, Uchihara T (2008). Necrotizing fasciitis caused by Pseudomonas aeruginosa. *Internal Medicine*.

[10] Lo WT, Cheng SN, Wang CC, Chu ML (2005). Extensive necrotising fasciitis caused by Pseudomonas aeruginosa in a child with acute myeloid leukaemia: case report and literature review. *European Journal of Pediatrics*.

[11] Childers BJ, Potyondy LD, Nachreiner R (2002). Necrotizing fasciitis: a fourteen-year retrospective study of 163 consecutive patients. *American Surgeon*.

[12] Hsieh T, Samson LM, Jabbour M, Osmond MH (2000). Necrotizing fasciitis in children in eastern Ontario: a case-control study. *Canadian Medical Association Journal*.

[13] Frank G, Mahoney HM, Eppes SC (2005). Musculoskeletal infections in children. *Pediatric Clinics of North America*.

